# CRISPR/Cas9-mediated knockin of IRES-tdTomato at Ins2 locus reveals no RFP-positive cells in mouse islets

**DOI:** 10.1007/s10142-023-00960-1

**Published:** 2023-01-18

**Authors:** Xueling Zhou, Qi Fu, Tao Yang, Min Sun

**Affiliations:** 1 Department of Endocrinology, The First Affiliated Hospital of Nanjing Medical University, Nanjing, 210029 China; 2 Department of Endocrinology, Affiliated Zhongda Hospital of Southeast University, School of Medicine, Southeast University, Nanjing, China

**Keywords:** CRISPR/cas9, tdTomato, Ins2, C57BL/6mice

## Abstract

Using the CRISPR/Cas9 genomic editing technology, we constructed a transgenic mouse model to express specific fluorescent protein in pancreatic β cells, which harbor tdTomato exogenous gene downstream of the Ins2 promoter in C57BL/6 J mice. The Ins2-specific single-guide RNA-targeted exon2 was designed for the CRISPR/Cas9 system and Donor vector was constructed at the same time. Then Cas9, sgRNA, and Donor vector were microinjected in vitro into the mouse zygotes that were implanted into pseudo-pregnant mice. We obtained homozygotes through mating heterozygotes, and verified the knockin effect through genotype identification, in vivo imaging, and frozen section. Six F0 mice and stable inherited Ins2-IRES-tdTomato F1 were obtained. Genome sequencing results showed that the knockin group had no change in the Ins2 exon compared with the control group, while only the base sequence of tdTomato was added and no base mutation occurred. However, in vivo imaging and frozen section did not observe the expression of red fluorescent protein (RFP), and the protein expression of knockin gene tdTomato was negative. As a result, the expressions of tdTomato protein and fluorescence intensity were low and the detection threshold was not reached. In the CRISP/Cas9 technique, the exogenous fragment of IRES connection would affect the transcription level of the preceding gene, which in turn would lead to low-level expression of the downstream gene and affect the effect of gene insertion.

**Supplementary information:**

The online version contains supplementary material available at 10.1007/s10142-023-00960-1.

## Introduction

Whether type 1 or type 2 diabetes, pancreatic β cell dysfunction is central to the pathogenesis (Tan et al. 2019). Therefore, the key to prevention and control of diabetes is to study the function and regulation mechanism of islet β cell (Fu et al. 2013; Tan et al. 2019). Currently, islet β cell lines (e.g., MIN6 and INS-1) and primary islets are commonly used in vitro. However, insulinoma cell lines differ from the primary islet β-cells by their morphology, level of differentiation, insulin content, and most importantly by their abnormal response to glucose stimulation (Douillet et al. 2013). It has been shown that MIN6 is not a pure beta cell line but a mixed cell line with other pancreatic endocrine hormones, including not only insulin-positive cells but also insulin and glucagon or somatostin double-positive cells (Nakashima et al. 2009). Although primary islets can better reflect the normal physiological function of pancreatic β cells and insulin-secreting cells, it is impossible to study β cells alone when cultured as a whole because islets of Langerhans are a compact mass of multiple cells. As further studies have been performed, growing evidences have proved that there is a great degree of heterogeneity among β cells in glucose responsiveness, insulin secretion, proliferation ability, and maturation status, which can be divided into subtypes (Roscioni et al. 2016). β cell dedifferentiation and transdifferentiation also represent alternative sources of β cell heterogeneity, which can affect the development and treatment of diabetes (Chen et al. 2019). All the above studies require to be observed at a single-cell level.

In recent years, many studies have attempted to use primary beta cells as research objects (Krogvold et al. 2015), but primary β cells of high purity and activity are difficult to extract from tissues as a homogeneous population. Common methods for obtaining primary islet β cells (e.g. flow cytometry sorting (Stangé et al. 2003; Akbarzadeh et al. 2008), immunomagnetic bead sorting (Tamagno et al. 2014), Raman spectroscopy sorting (Rong et al. 2018)) have many defects, including the low efficiency of isolation and the damages of cellular structure and viability during isolation, which are not suitable for long-term cell culture and intervention in vitro. Thus, it is quite crucial to obtain sufficient, healthy, and stable primary islet β cells for diabetes treatment and research.

Gene editing is a technique for fixed-point modification of DNA nucleotide sequences, which can precisely delete, insert, and substitute the nucleotide sequence of targeted DNA fragments, a powerful method for studying gene function and producing genetically modified animals (Wang et al. 2016). The CRISPR/dCas9-based gene editing technology is mediated by an interaction between guide RNA (gRNA) and target DNA, which has quickly become popular in laboratories all over the world and become a powerful helper for biological research owing to its advantages of low cost, convenient operation, and high efficiency (Cress et al. 2016; Himeda et al. 2016; Luo et al. 2021). CRISPR/Cas9 is a third-generation technology for site-directed genome editing, following zinc finger nucleases (ZFN) and transcription activator like effector nucleases (TALEN), known as the magic scissors of gene editing. In contrast to other gene editing techniques, the biggest breakthrough of this technology is that it can edit not only a single gene, but also many genes at the same time (Liu et al. 2016; Luo et al. 2021), which has brought light to primary cell isolation for islet. Therefore, our study used CRISPR/Cas9 technology to construct transgenic mice to express specific fluorescent protein tdTomato in pancreatic β cells for extracting high-purity primary beta cells, and if successful, we can exploit fluorescence intensity changes of β cells in response to cellular heterogeneity so as to perform primary screening on various subtypes. As an optimized red fluorescent protein (RFP), tdTomato is generally brighter and more photostable than green fluorescent protein (GFP) (Shaner et al. 2007; Bianchi et al. 2015). We make Ins2-IRES-tdTomato mice via the CRISPR/Cas9 system. Cas9 mRNA, sgRNA, and donor will be co-injected into zygotes. SgRNA direct Cas9 endonuclease cleavage on exon2 near the stop codon (TGA) and create a DSB (double-strand break). Such breaks will be repaired, and result in IRES-tdTomato inserted before the stop codon by homologous recombination. The pups will be genotyped by PCR, followed by sequence analysis.

## Materials and methods

### Materials

Mice in C57BL/6 J background were used in this study, and pseudopregnant surrogates were ICR females, which were all SPF grade. The Model Animal Research Center of Nanjing University was commissioned to complete gene knockin and breeding. All of the animal experiments were approved by the Nanjing Medical University Ethics Committee (IACUC-1901045). Various restriction enzymes, T4 DNA ligase, and HiScribe T7 Quick High Yield RNA Synthesis Kit were purchased from NEB Corporation (Ipswich, MA, USA). Trizol reagent and Reverse Transcription Kit were purchased from TaKaRa (Otsu, Japan). RNA transcript was in vitro synthesized by mMESSAGE mMACHINE® T7Ultra Kit (ThermoFisher) and RNAs were purified using Ambion MEGAclear Kit (Ambion).

PCR primers were synthesized by Genscript Biotechnology Co. Ltd, and DNA sequencing reactions were done by Hefei General Biotechnology Co. Ltd. (Hefei, China). See Table 1 for gRNA sequences.

**Table 1:** Sequence list of gRNAs

sgRNA name	The sequences of gRNAs (5’ → 3’)	PAM
sgRNA1	CCTTCAGACCTTGGCACTGG	AGG
sgRNA2	AGCAGGTGACCTTCAGACCT	TGG

## Methods

### CRISPR/Cas9 site design

**Fig. 1:**
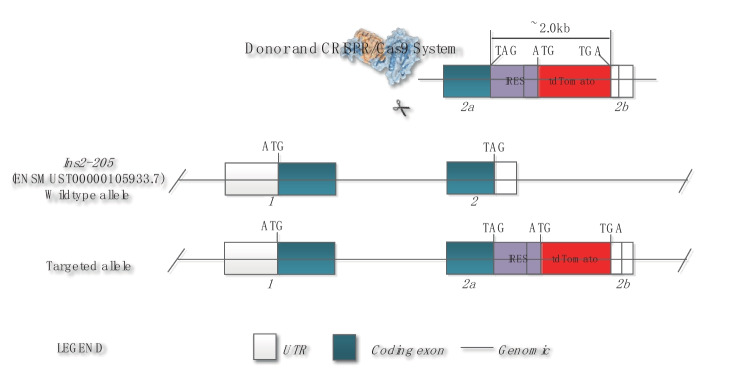
CRISPR/Cas9-mediated knockin strategy of IRES-tdTomato

**Fig. 2:**
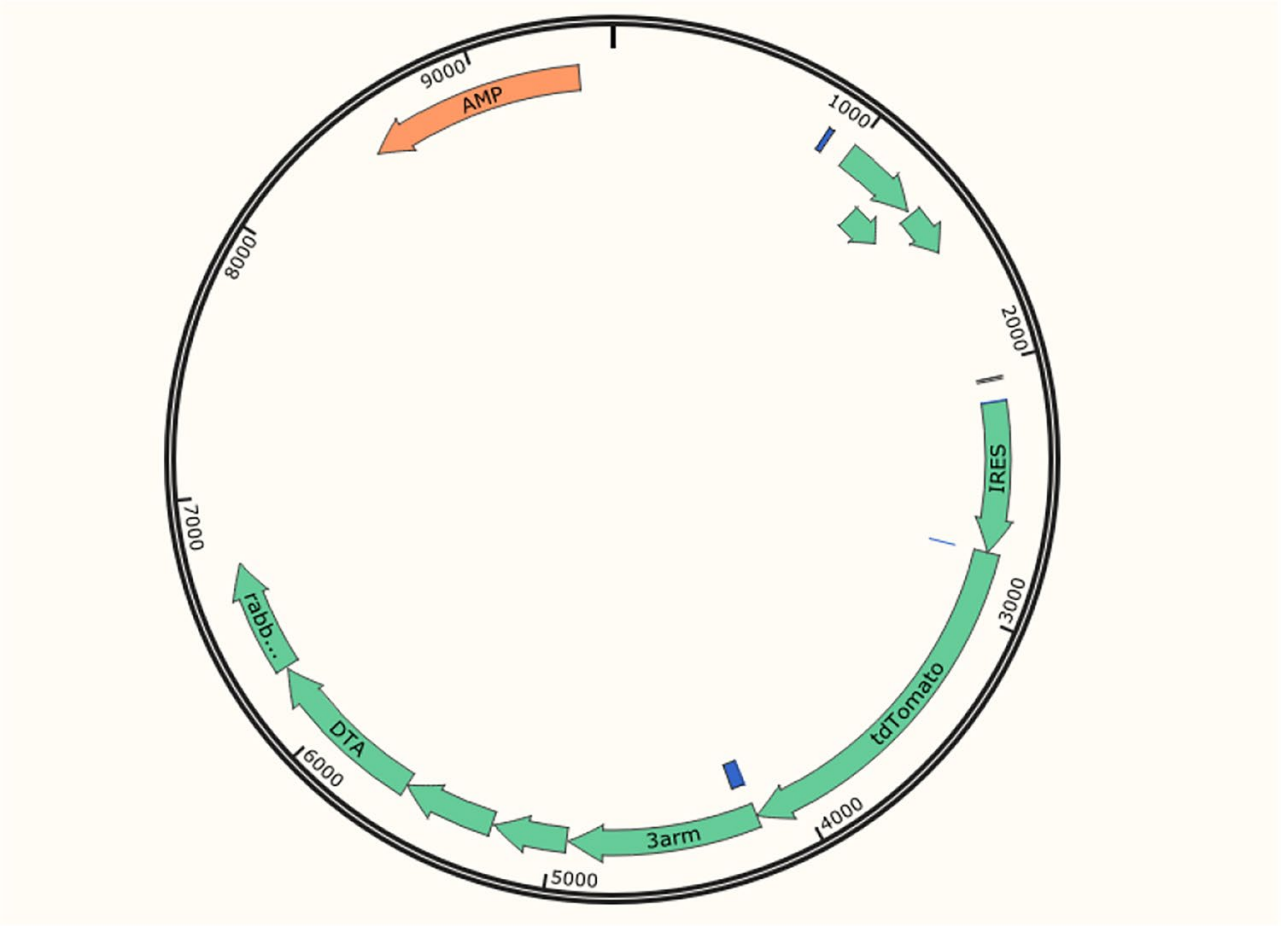
Schematic diagram of the targeting vector structure

Transcript *Ins2-205*(ENSMUST00000105933.7) is selected for presentation of the recommended strategy. *Ins2-205* gene has 2 exons, with the ATG start codon in exon1 and TGA stop codon in exon2. The IRES-tdTomato was then inserted after the translation end site TAG of Ins2-205 of the Ins2 transcript, shown in Fig. 1. The primer sequences used for the construction of the sgRNA expression plasmids are listed in Table 1, and Cas9 protein was directed to cut DNA double strand at specific sites of sgRNA-guided sequence target, shown in Fig. 2.

### In vitro transcription of Cas9 mRNA and guide RNAs

Cas9 mRNA was transcribed in vitro with mMESSAGE mMACHINE ® T7 Ultra Kit. Guide RNA (gRNA) was synthesized using HiScribe T7 Quick High Yield RNA Synthesis kit. Transcribed products were purified with Ambion MEGAclear Kit.

### Generation of the tdTomato knockin mice

We make *Ins2-IRES-tdTomato* mice via the CRISPR/Cas9 system. Cas9 mRNA, guide RNAs, and donor vector were microinjected into zygotes (C57BL/6 J). sgRNA would direct Cas9 endonuclease cleavage on exon2 near the stop codon (TGA) and create a DSB (double-strand break). Such breaks would be repaired, and result in *IRES-tdTomato* inserted before the stop codon by homologous recombination. After injection, surviving zygotes were immediately transferred into pseudopregnant ICR females and the pups born were called F0 generation mice.

### Ins2-IRES-tdTomato mouse breeding and genotype identification

F0 mice were mated with C57BL/6 J mice to obtain heterozygous germline F1 offspring. The genotypes were confirmed by PCR and DNA sequencing (see Supplementary Table 1). Transgenic positive heterozygous germline mice were interbred to obtain homozygous mice.

### In vivo fluorescence imaging

Ins2-IRES-tdTomato mice were observed by an fluorescent in vivo imaging system (SI AmX1, Spectral Instruments Imaging, USA), which were anesthetized with isoflurane (2.5% isoflurane for induction and 0.1% for maintenance).

### Frozen sections and immunofluorescence

Pancreatic tissues were frozen embedded in O.C.T. Compound (Sakura). Frozen tissues were sectioned at 5–6-μm thickness, which were incubated with the primary antibody: insulin (AF1159; Biyuntian). After incubation with the fluorescence-conjugated secondary antibody Alexa Fluor 488 goat anti-rabbit IgG (Jackson Immunoresearch), the cell nuclei were stained with DAPI. Digital images were captured using Zeiss fluorescence microscope.

### Real-time quantitative PCR (RT-qPCR)

Total RNA was extracted using TRIZOL®/Chlorophorm/Phenol method, and reverse-transcribed using the PrimeScriptTM RT Master Mix (TaKaRa, Japan). Real-time PCR was conducted on the StepOnePlus Real-Time PCR System (Thermo Fisher) with Hieff® qPCR SYBR-Green Master Mix (Yeasen, Shanghai, China). The relative expression of each target gene was normalized using murine β-actin as a reference gene and represented as 2^ΔCt^ for each sample or as fold changes (2^ΔΔCt^) relative to the control. See Supplementary Table 2 for primer sequences.

### Oral glucose tolerance test (OGTT) and glucose-induced insulin release test

Five mice at 13 weeks old from the Ins2-IRES-tdTomato mouse group and wild-type background mouse group were randomly selected, respectively. Oral glucose tolerance was assessed in overnight-fasted mice by measuring tail blood glucose 0, 15, 30, 60, and 120 min after oral administration of 2 g/kg glucose by gavage. Plasma samples were collected at 0, 30, and 120 min for insulin measurement.

### Statistical analysis

Real-time quantitative PCR results were from three repeated independent experiments, and each experimental group comprised a minimum of five animals. Statistical analysis was performed with SPSS statistical software package, version 24.0, and measurement data were expressed as mean ± standard deviation (x̅ ± *s*). Two-tailed Student’s *T* test or analysis of variance (ANOVA) was used to compare two groups or multiple groups, respectively. In all statistical analyses, a *P*-value < 0.05 was considered statistically significant. Statistical charts were drawn using GraphPad Prism 6.0e software.

## Results

### The acquisition and identification of Ins2-IRES-tdTomato transgenic mice

#### F0 generation acquisition and identification

**Fig. 3:**
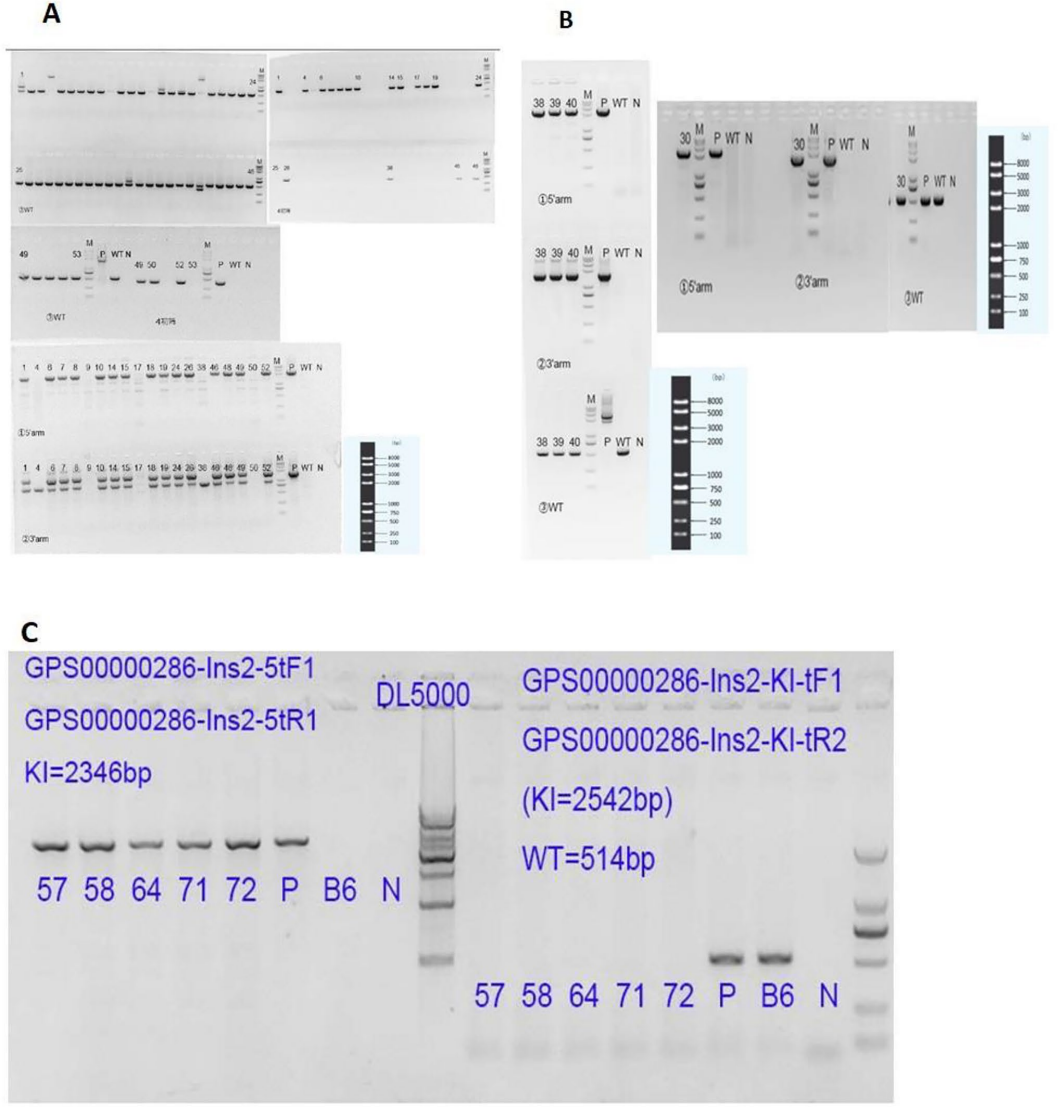
Electrophoresis graph of PCR products. (**A**) Mice nos. 1, 6, 7, 8, 15, 18, and 26 were positive F0 generation mice. (**B**) Mice nos. 30, 38, 39, and 40 mice were positive F1 generation mice. Numbers were mouse tail number, P was positive control, WT was wild type C57BL/6 J, N was negative blank control, and M was DNA marker. (**C**) Mice nos. 57, 58, 65, 71, 72, and 18 mice were all Ins2-IRES-tdTomato Ki/ki; B6, wild type C57BL/6 J; N, no template control. DL5000Marker:5000 bp/4000 bp/3000 bp/2500 bp/2000 bp/1500 bp/1000 bp/500 bp

**Fig. 4:**
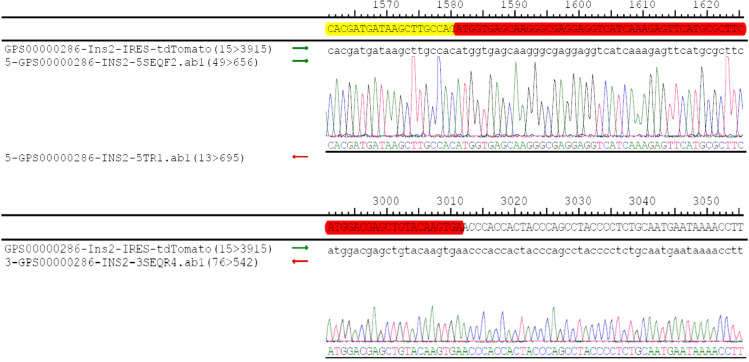
Partial genomic DNA sequences of Ins2-IRES-tdTomato transgenic mice. The base sequence of tdTomato was from 1581 to 3011 in the sequencing peak map

Fifty-three F0 mice were born by microinjection and transplantation of fertilized zygotes. As shown in Fig. 4A, nos. 1, 6, 7, 8, 15, 18, and 26 were identified by PCR testing as 7 positive F0 generation mice, with a positive rate of 13.21%. One died (no. 6) and 6 mice (3 males and 3 females) were left before cage separation. A total of three pairs of primers were designed to amplify the transgenic sequence. (1) The upstream and downstream sequences of the primers were located on ins2 and tdTomato, respectively, and the size of the amplification product was 2346 bp. (2) The upstream and downstream sequences of the primers were located on tdTomato and ins2, respectively, and the size is 1656 bp. (3) The upstream and downstream sequences of the primers were all located on ins2, and the size of the amplified products is WT514 bp.Transgene-positive mice were identified by PCR reaction of primers ① and ② as shown in Fig. 3A. Six transgene-positive mice were verified by sequencing of the DNA from tail snips. The insertion position was accurate. Compared with the wild type, the exons have not changed, but the base sequence of tdtomato has been increased, from 1581 to 3011 in the sequencing peak map (see Fig. 4). The total size of tdTomato was 1431 bp, the base sequence of tdTomato was completely consistent with the reference sequence, and there was no base mutation.

#### Acquisition and identification of F1 generation mice and homozygous offspring mice

The positive F0 mice were crossed with C57BL/6 J mice to obtain F1 generation heterozygotes. After PCR and sequencing, 30#, 38#, 39#, and 40# mice were positive F1 generation mice, as shown in Fig. 3B. Positive heterozygous ki/wt mice were mated to generate homozygous Ki/ki offspring, and PCR electrophoresis identification of some homozygous progeny is shown in Fig. 3C.

### In vivo imaging of Ins2-IRES-tdTomato transgenic mice

**Fig. 5:**
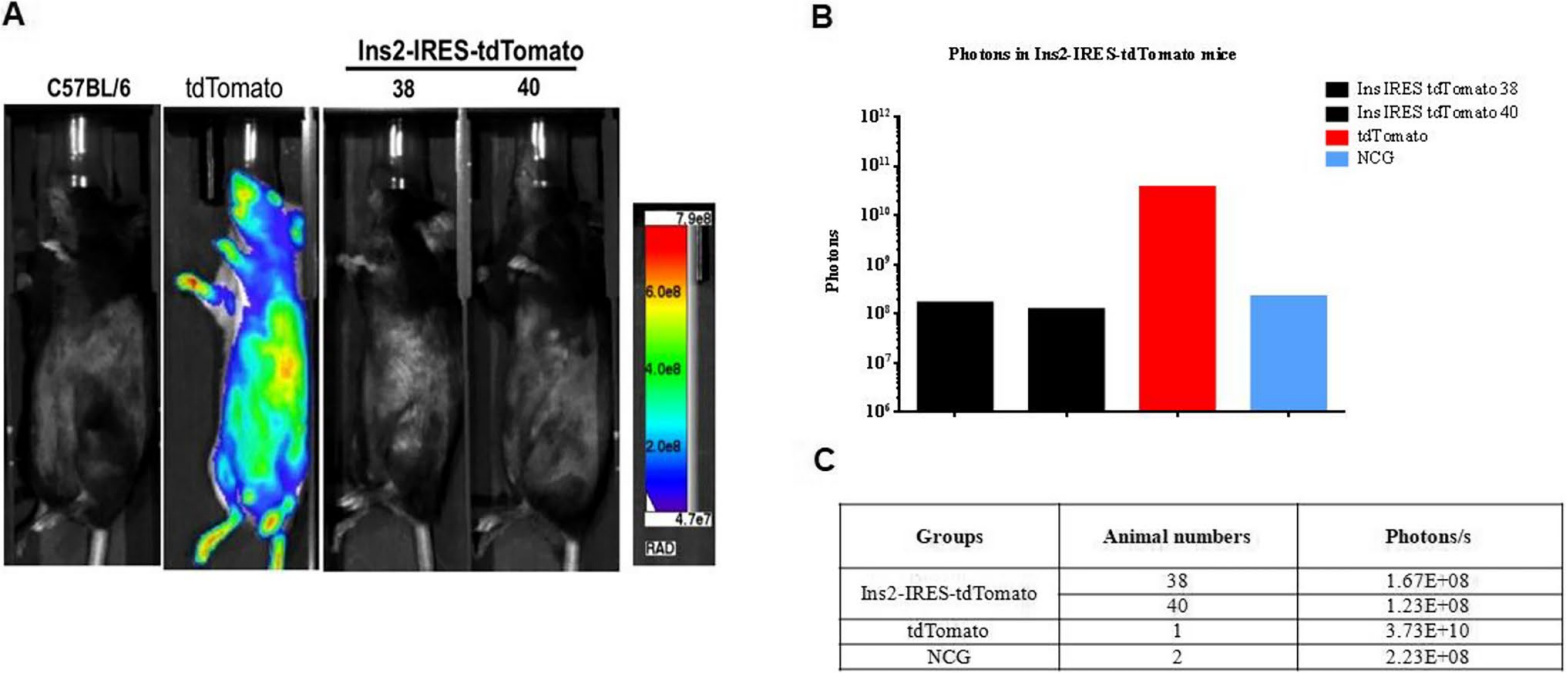
In vivo imaging of Ins2-IRES-tdTomato heterozygous mice. (**A**) Imaging pictures. (**B**) Luminescence area. (**C**) Total photon number; C57BL/6 J wild-type mouse was used as normal control group (NCG), tdTomato as positive control

**Fig. 6:**
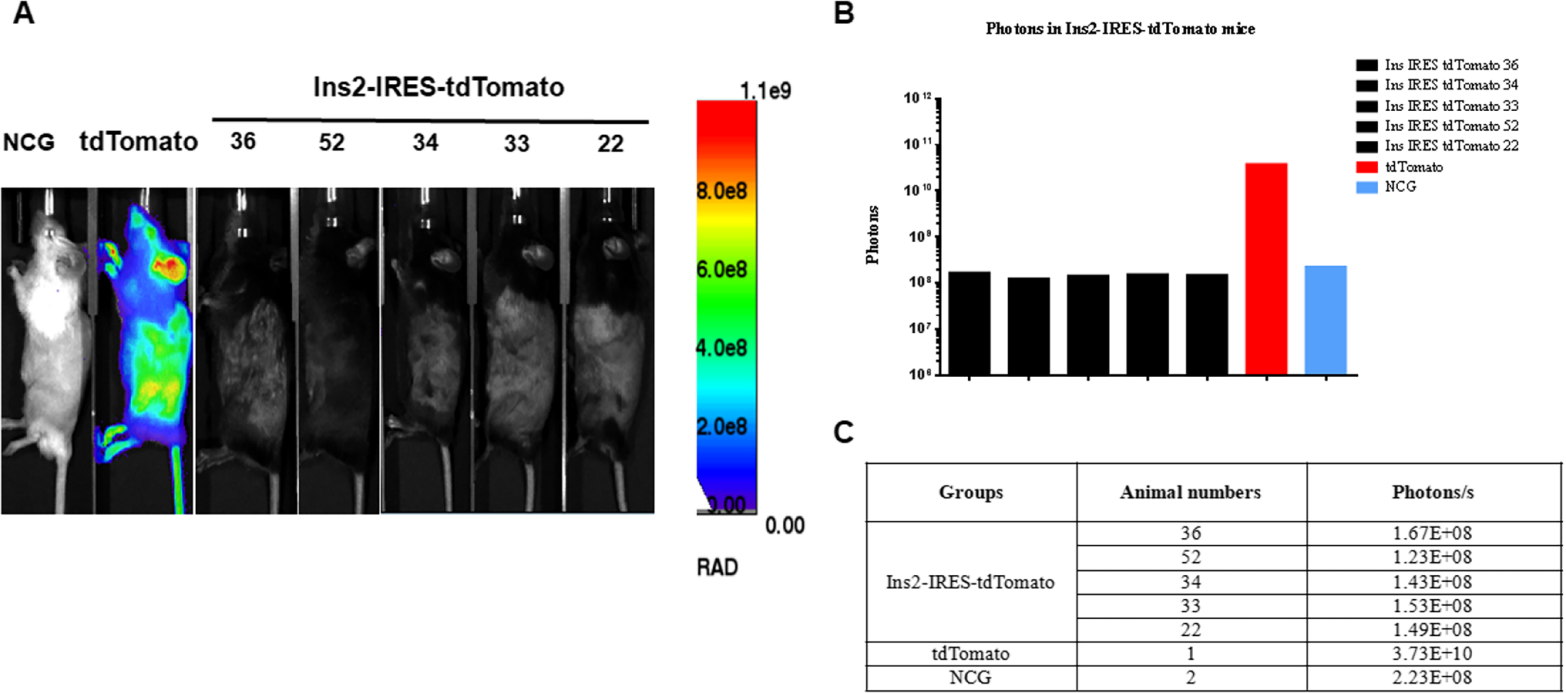
In vivo imaging of Ins2-IRES-tdTomato homozygous mice. (**A**) Imaging pictures. (**B**) Luminescence area. (**C**) Total photon number; NCG was used as wild-type C57BL/6 J negative control, tdTomato as positive control

In order to detect the expression of tdTomato, the gene-identified positive heterozygous F1 generation No. 39 and No. 40 were imaged live. Results showed that an obvious fluorescent signal was detected in the systemic expression of tdTomato mouse as positive control, and no obvious fluorescent signal was detected in wild-type C57BL/6 J mouse as negative control. But no tdTomato fluorescent signal was detected in the pancreatic tissue of Ins2-IRES-tdTomato mice (see Fig. 5). F1 were mated to generate homozygotes nos. 22, 33, 34, 36, and 52, which were observed by in vivo imaging. As shown in Fig. 6, tdTomato fluorescent signal was also not detected in Ins2-IRES-tdTomato homozygotes.

### Immunofluorescence staining of frozen sections in Ins2-IRES-tdTomato mice

**Fig. 7:**
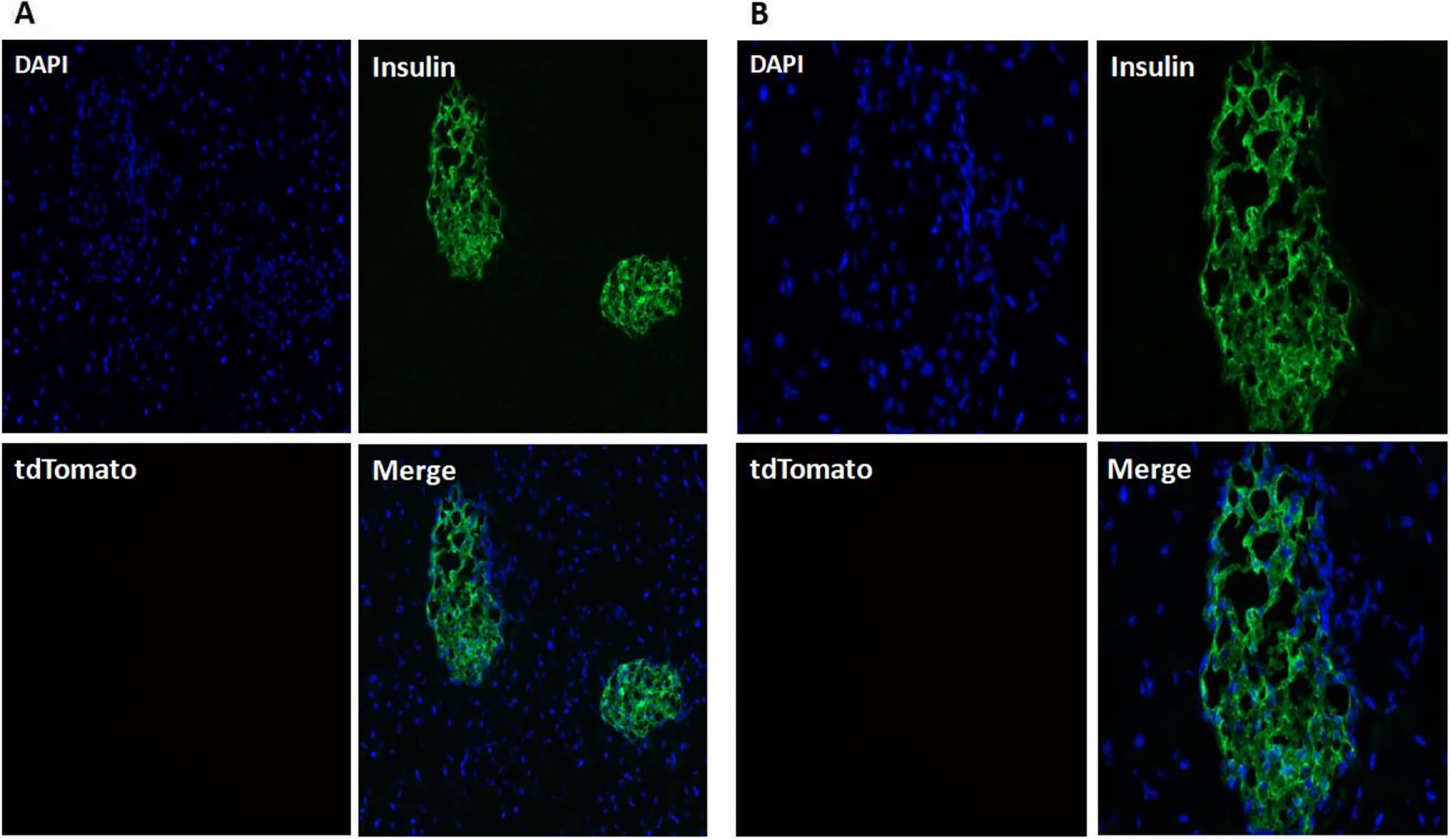
Immunofluorescence staining of pancreas frozen sections to detect tdTomato fluorescent signal. (**A**) Observation under 100 × fluorescence microscope. (**B**) Observation under 400 × fluorescence microscope

In order to clarify the expression and localization of tdTomato in islets of transgenic mice, immunofluorescence staining of insulin was performed on frozen pancreatic sections. The results of co-expression with insulin are shown in Fig. 7, which shows that there is no tdTomato fluorescent signal in insulin-positive cells. So far, this suggested that Ins2-IRES-tdTomato transgenic mice were negative.

### Quantitative real-time PCR (qRT-PCR) assay of Ins1, Ins2, and tdTomato in islets

**Fig. 8:**
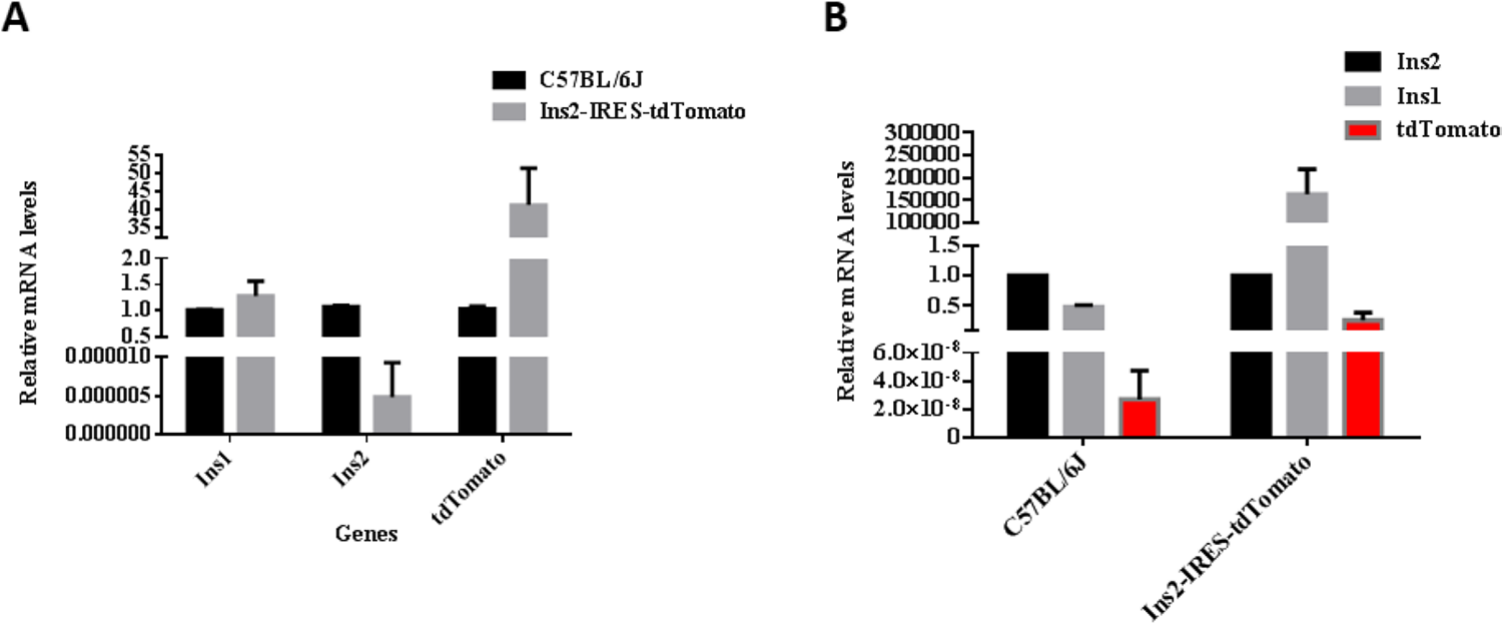
mRNA level of Ins1, Ins2, and tdTomato in the islets of Ins2-IRES-tdTomato mice and C57BL/6 J mice. (**A**) Comparison of relative gene expression between the two groups. (**B**) Gene expression of three target genes in each group

In order to detect the expression of Ins1, Ins2, and tdTomato in Ins2-IRES-tdTomato homozygous mice, qRT-PCR was used to measure the expression of the three genes above in this study. Figure 8A compares relative gene expression between the two groups. Wild-type C57BL/6 J mice were used as the control group: the expression of Ins1 in transgenic mice was 1.278 ± 0.0.284 times higher than that in the control group (*P* > 0.05), the expression of Ins2 was less than 1/10,000 times that of the control group, and the expression level of tdTomato was 41.10 ± 10.21 times higher than that in the control group. Figure 8B shows gene expression of three target genes in each group. In wild-type C57BL/6 J mice, the expression of Ins1 was 0.481 ± 0.0344 times that of Ins2, and tdTomato was almost not expressed, which was 1/10,000,000 times that of Ins2. In Ins2-IRES-tdTomato mice, Ins1 was more than 100,000 times that of Ins2, and the expression level of tdTomato was 0.261 ± 0.124 times that of Ins2. The major results can be summarized as follows: (1) The expression profiles of ins2 and tdTomato in Ins2-IRES-tdTomato mice were basically consistent, indicating that the inserted position was correct and the transcription of exogenous gene could be initiated. However, Ins2 expression was inhibited compared with the control group, and tdTomato expression was low at the transcriptional level. (2) The expression of Ins1 in transgenic mice was slightly higher than that in wild-type C57BL/6 J mice, but there was no statistical difference (*P* > 0.05).

### Oral glucose tolerance test and glucose-induced insulin release test

**Fig. 9:**
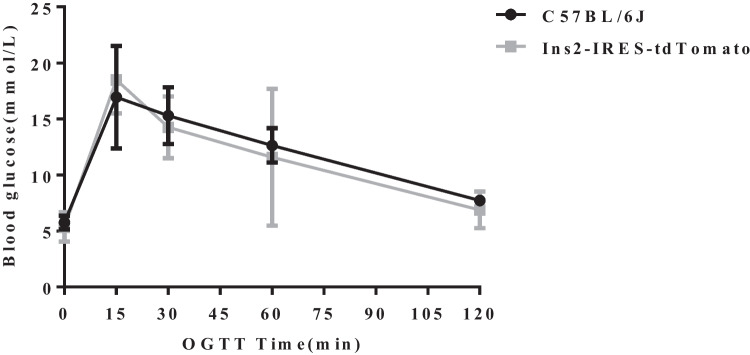
Oral glucose tolerance test at 13 weeks old

**Fig. 10:**
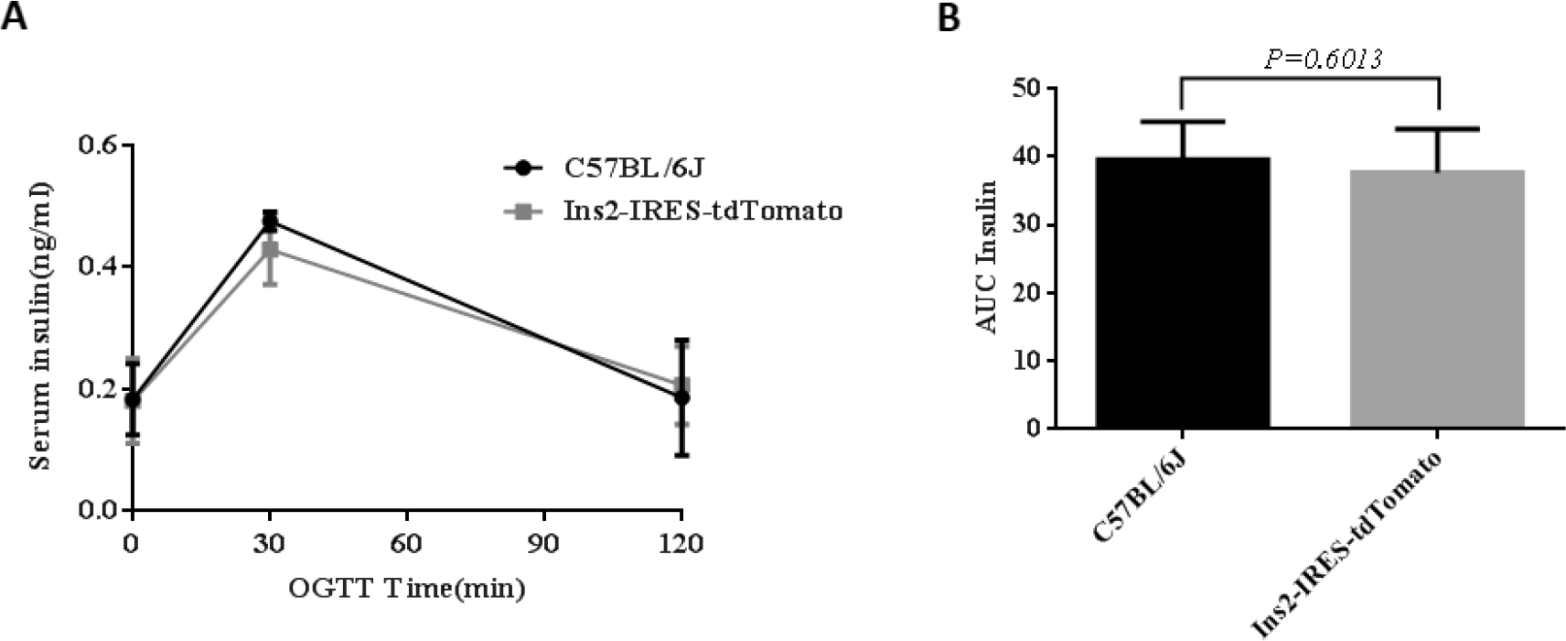
Glucose-induced insulin release test during OGTT at 13 weeks old. (**A**) Insulin curve. (**B**) Area under the curve of insulin

To verify whether knockin tdTomato has an effect on islet function, oral glucose tolerance test and insulin release test were performed between male transgene-positive homozygous mice and wild-type mice all at 13 weeks old, and the results showed no statistically significant differences (see Figs. 9, 10).

## Discussion

In many respects, the study of diabetes is inseparable from studying pancreatic beta cells at the single-cell level. In recent years, CRISPR/Cas9 fixed-point editing gene technology has become popular worldwide, which enables efficient gene editing without the time and expense necessary to cross the transgenic animals flanked by loxP sites with Cre recombinase-expressing lines. Moreover, CRISPR/Cas9 could achieve multiplex genome editing (Wang et al. 2016; Mollanoori and Teimourian 2018). Therefore, we used CRISPR/Cas9 technology to generate transgenic mice to express specific fluorescent protein tdTomato in pancreatic β cells for extracting high-purity primary beta cells. The dysfunction and death of insulin-producing pancreatic β cells are key elements in the pathogenesis of type 1 and type 2 diabetes (Duan et al. 2016).

The precursor of insulin is highly conserved among different species (Li et al. 2016), but unlike most mammals, mice and rats have two non-allelic insulin genes, *Ins1* and *Ins2*, which encode proinsulin I and II, respectively (Schoeller et al. 2012; Li et al. 2016). Ins1 arose from a duplication of the ins2 ancestral gene ∼20 million years ago and has since been retained in the mouse and rat genome (Shiao et al. 2008). These two genes have different genomic structures, but both are simultaneously expressed in all β cells at a ratio of 1:2 at the mRNA and protein levels (Li et al. 2016). Experiments have shown that Ins1 or Ins2 knockout in mice does not lead to abnormal development and metabolic disorders, which is likely due to compensatory increase in gene transcription (Duvillié et al. 1997; Leroux et al. 2001). Moreover, murine ins2 is orthologous to the human insulin gene (Schoeller et al. 2012; Li et al. 2016). So these results provide strong evidence for targeted Ins2 genetic manipulation in vivo.

Other studies have shown that the traditional Cre-loxP system has been used to knockin the tagged genes for pancreatic β cells expressing specific marker proteins (Li et al. 2016). In order to employ the Cre/loxP system in organisms, the loxP sites need to be introduced beforehand at the anticipated genomic position (Lansing et al. 2020), which is a tedious process, and the target gene of loxP sites is activated. By Cre recombinase rather than directly controlled by the Ins2 promoter. Considering CRISPR/Cas9 can be used to edit multi-gene in the other major islet endocrine cell types for the follow-up study. So we adopted the CRISPR/Cas9 system for gene editing in pancreatic β cells. The exogenous gene (tdTomato tag) was introduced into the non-coding region of Ins2 exon via the CRISPR/Cas9 technology, which was linked to Ins2 with an IRES sequence, and under the Ins2 promoter control, the genes before and after the IRES can be co-transcribed, but translated independently. We constructed the transgenic mouse model to express specific tdTomato protein in pancreatic β cells and red fluorescent protein was used as the β cell screening marker. How to prove whether the model is successful? We have identified the expression of the knockin gene tdTomato in the Ins2-IRES-tdTomato–positive model mouse, and compared it with the background mouse in terms of metabolic phenotype, islet function, etc.

The transgenic mouse model with specific fluorescent labeling of pancreatic β cells was commissioned by the Model Animal Research Center of Nanjing University. It can be seen from the results that a total of 53 mice were obtained after microinjection of fertilized eggs and embryo transfer. Detected by PCR and further confirmed by sequencing, a total of 7 positive mice F0 were obtained; 1 died later. The remaining three male mice were numbered 1, 15, and 26, and the three female mice were numbered 7, 8, and 18. The gene editing efficiency was 13.2%. Relevant studies have shown that the efficiency of homology-directed repair (HDR) by CRISPR/Cas9 technology is low, only 3.5 to 15.6% (Hruscha et al. 2013; Mali et al. 2013), which is roughly consistent with our experimental efficiency, and according to the sequencing results, there is no off-target effect, indicating that the sgRNA we designed based on the conserved “identity information”-protospacer adjacent motif (PAM) works better. At the same time, the HDR efficiency is slightly higher than the average efficiency abroad. F1 generation mice were obtained by natural breeding of gene editing positive mice F0 and wild-type mice (C57BL/6 J), which were identified by PCR and sequencing, and the results showed that four F1 generation mice numbered 30, 38, 39, and 40 were positive, among which two numbered 30 and 40 were female and the other two were male. Moreover, the sequencing results also showed that the four positive mice only increased the base sequence of tdTomato, and the alignment was completely consistent with the standard sequence, indicating that the genetics was relatively stable and there was no base mutation. At the same time, the Ins2 exons did not change compared with the wild type. Four F1-positive mice were then self-bred to produce positive heterozygous and homozygous offspring.

In order to explore the actual expression of the Ins2-IRES-tdTomato gene in the tdTomato knockin mouse line, whereas gene transcription is one aspect, post-transcriptional translation is another (Watada et al. 2000). We first consider intuitive detection live in vivo imaging. The tdTomato gene can auto-fluoresce red under the excitation light source. Two heterozygotes numbered 38 and 40 and five homozygotes numbered 22, 33, 34, 36, and 52 were subjected to in vivo imaging detection in batches. Unfortunately, it was found that no red fluorescence signal was detected in two gene-identified positive homozygous Ins2-IRES-tdTomato mice and five gene-identified positive homozygous Ins2-IRES-tdTomato mice. For this reason, we performed frozen sections and insulin immunofluorescence staining of gene-identified positive mice, and the results showed that no red fluorescent signal was detected in insulin-positive cells. So far, it shows that the tdTomato gene is not expressed or the expression level is very weak in islet β cells, and the instrument cannot detect it basically. For this reason, we also carried out frozen section and immunofluorescence staining for insulin localization in the transgene-positive mice, and the results showed that no red fluorescence signal was detected in the insulin-positive cells. Thus, tdTomato protein was not expressed in islet β cells, or too weak to be detected out by the instrument. The Ins2-IRES-tdTomato gene we designed is transcribed by the promoter of Ins2 to the simultaneous action of Ins2 and tdTomato gene, so it is necessary to know the expression of Ins2 gene in islet β cells. We used qPCR to determine the expression of Ins1 and ins2, and tdTomato gene transcription levels in the islets of Ins2-IRES-tdTomato homozygous mice with positive gene identification. We used qPCR to determine the expression of Ins1, Ins2, and tdTomato genes in islets of Ins2-IRES-tdTomato homozygous mice. The detection and identification results show that (1) the transcription level of tdTomato in Ins2-IRES-tdTomato mice is about 26% of the Ins2 transcription level, which also confirms that the previous sequencing results indicate that the expected position was inserted and the transcription of exogenous genes was started. Previous studies have also shown that the expression of the IRES-dependent second gene ranged from 6 to 100% (in most cases between 20 and 50%) that of the first gene (Mizuguchi et al. 2000), which is roughly consistent with our experimental efficiency. However, the transcription level of Ins2 in transgenic mice was only very weak, equivalent to 1/10,000 of that in the control group. Compared with wild mice, the expression of Ins2 was inhibited, so the transcription level of tdTomato is also low. (2) The knockin tdTomato gene did not affect the expression of Ins1 gene, and the expression of Ins1 in transgenic mice was slightly higher than that in wild-type C57BL/6 J mice, between which there was no statistical difference. In the metabolic phenotype analysis, it was found that there was no difference in the results of glucose tolerance test and insulin release test between transgenic positive homozygous mice and wild-type mice at the same age, and no effect on pancreatic islet function, indicating that Ins1 gene was compensatively upregulated while Ins2 gene expression is reduced or not expressed after editing. Thus, the glucose metabolism of transgenic mice tended to be normalized, which was consistent with Leroux L et al. (2001). To analyze the negative reasons of transgenic mice, we should first consider whether the site selection of Ins2-IRES-tdTomato gene knockin is inappropriate.

It was found that when some researchers used the Cre recombinase-loxP (Cre-Lox) system for editing Ins2, they constructed the Ins2-IRES-Cre expression system by inserting IRES-Cre-pA after the exon of Ins2 to generate Ins2-Cre knockin mice, which crossed with Rosa26-tdTomato mice. After Cre recombinase cuts the stop codon, tdTomato red fluorescence can be expressed in the islets, and the transcriptional expression of Ins2-IRES-Cre in other mouse tissues can be detected (Li et al. 2016). This is the same as the gene knockin site we used in Ins2-IRES-tdTomato, but the difference is that we used CRISPR/Cas9 gene editing technology and the principle of homologous recombination for the first time to genetically modify the target site, which shows that there is no error in the fundamentals of our study. The widely used linking elements in gene editing are 2A and IRES. Since the 2A element is a polypeptide, it will leave approximately 20 amino acids on the tail of the previous protein (Chng et al. 2015), which would inevitably affect the small molecule Ins2 while the IRES element does not participate in transcription and translation, and the two proteins can be translated independently without modification (Mizuguchi et al. 2000). Literature suggests both the capacity of IRES and the translation efficiency should be taken into consideration when constructing the IRES-dependent gene expression. If the same levels of expression are desired of the first and second genes, decreased efficiency of the translation of the first gene by non-Kozak sequence or a change in the ATG start codon would be possible strategies (Li et al. 1997). It has been demonstrated that in a bicistronic construct with IRES, the capacity of IRES-dependent second gene expression is usually significantly lower than that of cap-dependent first gene expression. This information must be taken into account for the use of IRES in gene transfer and gene therapy experiments (Mizuguchi et al. 2000). However, the expression levels of the knockin tdTomato and Ins2 were both low in this study. The possible reason is that the IRES element and the translation efficiency of the gene of interest are not optimized (Mizuguchi et al. 1996, 1998). Therefore, it is considered that the insertion of exogenous fragments (IRES-tdtomato) affects the transcriptional regulatory element of Ins2, or the stability of the transcribed mRNA, and some mRNAs are degraded, which affects the transcription level of Ins2, resulting in the extremely low transcription level of tdTomato and the failure of fluorescent protein expression. In addition, it is not possible to rule out that CRISPR/Cas9 gene editing technology is selective for insertion sites.

In general, there is no problem in the construction of targeting vector and gene knockin technology in this experiment, and the results of PCR experiment and sequencing are also verified. The main consideration may be that the exogenous fragment (IRES-tdTomato) affects the transcription level of Ins2, which may be due to the fact that the designed Ins2-IRES-tdTomato gene has not been effectively optimized, thus showing poor transcription efficiency and resulting in too low expression of tdTomato for effective detection.

## Supplementary information

Below is the link to the electronic supplementary material.

Supplementary file1 (PDF 156 KB)

## Data Availability

This declaration is “not applicable.”
