# Supplementary material for: CRISPR/Cas9-mediated knockin of IRES-tdTomato at Ins2 locus reveals no RFP-positive cells in mouse islets
Source: Funct Integr Genomics. 2023 Jan 18;23(1):42. doi: 10.1007/s10142-023-00960-1 (PMC9849276; doi:10.1007/s10142-023-00960-1)
Supplement: Supplementary file 1 — Supplementary file1 (PDF 156 KB) [file 10142_2023_960_MOESM1_ESM.pdf]

Supplementary Table 1 Sequences of primers used to identify the genotype of transgenic mice

Identification sites	Primer name	Primer sequence (5'→3')	Product size
① 5'arm	F1	CAGCCCTAAGTGATCCGCTAC	WT:0bp
	R1	ATGACGGCCATGTTGTTGTCC	Targeted:2346bp
② 3'arm	F2	CCACCTGTTCCTGGGGCATG	WT:0bp
	R2	GGAACCTATCTAGCTGGAAGGAG	Targeted:1656bp
③ WT	F1	GGGTAGTAGGAGGTTGCTCAGC	WT:514bp
	R2	GTTCCAGGTTTGGTCTAACAGG	Targeted:2542bp

Supplementary Table 2 The sequences of gene primers were as follows:

Gene name	Forward and reverse primer(5'→3')	Tm（℃）
Mouse Tdtomato	F: GACTACACCATCGTGGAACAGTAC	60
	R: ATGACGGCCATGTTGTTGTC	60
Mouse Ins1	F: GAAGTGGAGGACCCACAAGTG	60
	R: ATCCACAATGCCACGCTT	60
Mouse Ins2	F:GAAGTGGAGGACCCACAAGTG	60
	R:GATCTACAATGCCACGCTTCTG	60
Mouse β-actin	F: GCTACAGCTTCACCACCACAG	60
	R: GGTCTTTACGGATGTCAACGTC	60
